# Renal Involvement in Children with Dengue Fever: A Study in Tertiary Care Hospital of Bangladesh

**DOI:** 10.1155/2020/4025267

**Published:** 2020-01-07

**Authors:** Azmeri Sultana, Jubaida Rumana, Smrity Roy, Seikh Farzana Sonia, Farhana Rahat, Ruma Parvin, Sharmin Afroze

**Affiliations:** ^1^Fellowship in Pediatric Nephrology (NUH, Singapore), Dr MR Khan Children Hospital and Institute of Child Health Affiliated by BCPS (Bangladesh College of Physician and Surgeon), BSMMU (Bangabandhu Sheikh Mujib Medical University), Singapore; ^2^Fellowship in Pediatric Nephrology (India), Asgar Ali Hospital, Dhaka, Bangladesh; ^3^Rangpur Medical College, Rangpur City, Bangladesh; ^4^Dr MR Khan Children Hospital and Institute of Child Health Affiliated by BCPS Bangladesh College of Physician and Surgeon, Bangabandhu Sheikh Mujib Medical University (BSMMU), Dhaka, Bangladesh

## Abstract

*Background and Objective*: Dengue has emerged globally as the most relevant viral infection transmitted by a mosquito bite and represents a major threat to public health. Dengue-related renal manifestations such as proteinuria, hematuria, acute kidney injury (AKI), and rhabdomyolysis are not uncommon, and acute kidney injury (AKI) is a serious complication of dengue fever. There is relatively few data on the renal manifestations of dengue fever in children. Hence, this study was conducted to evaluate the incidence, characteristics, and clinical outcome of dengue fever with renal manifestations. *Method*. This prospective cross sectional study was conducted in Dr. M R Khan Children Hospital and Institute of Child Health, Dhaka, over a period of 1 year from January 2018 to December 2018. The study was approved by the ethical committee of the institute. A total number of 316 patients were admitted with the diagnosis of dengue fever either NS1 positive or antibody IgM positive or both IgM and IgG positive. Data were collected in a structured questionnaire form and were analyzed by SPSS version 20.0. The disease severity was classified according to the World Health Organization criteria. Renal manifestations were divided into AKI groups using pRIFLE criteria. Proteinuria was defined as urinary protein >1+ (30 mg/dL) by dipstick test. Hematuria was defined as red blood cell (RBC) >5/*μ*L in a fresh uncentrifuged urine specimen. *Result*. Among 316 dengue patients, thirty-one patients (9.8%) had renal involvement. Most of the patients (54.83%) with renal manifestations were aged between 1 and 5 years. A total of 14 patients were found to have proteinuria (4.4%). Nephrotic-range proteinuria was seen in only one patient (0.3%). AKI was defined by pRIFLE criteria and was seen in 13 patients (4.1%); among AKI 6 (46.15%) had risk, three patients (23.07%) had injury and 4 (30.7%) had failure and needed peritoneal dialysis. Death occurred in 3 patients (9.6%) in dengue with AKI who had failure. The incidence of renal manifestations (proteinuria, hematuria, and AKI) is as high as 9.8% among patients with dengue, and those with AKI had significant morbidity and mortality. *Conclusion*. Renal involvement in children with dengue is not uncommon. Dengue associated with AKI had significant mortality and morbidity.

## 1. Introduction

Dengue infection has been identified as the fastest spreading mosquito-borne viral disease by World Health Organization [1]. It is an important febrile illness with wide spectrum of manifestations seen mainly in tropical countries [2]. WHO estimates 50–100 million cases of dengue in each year resulting in 24,000 deaths per year [3]. Dengue is a mosquito-borne disease, caused by serologically related but antigenically distinct single-strand RNA viruses; the viruses have been grouped into four serotypes (DENV-1, DENV-2, DENV-3, and DENV-4) belonging to the genus Flavivirus (family: Flaviviridae). *Aedes aegypti* is the primary mosquito vector; however, other species from the genus *Aedes*, such as *Aedes albopictus*, can also be vector of dengue virus transmission [4].

Incubation period of dengue virus infection is 3–14 days with a variety of clinical manifestation including asymptomatic infection, undifferentiated fever, dengue fever (DF), dengue hemorrhagic fever (DHF), and life-threatening dengue shock syndrome (DSS) [5]. Similar to other tropical infections, dengue infection is associated with multiple organ dysfunction involving liver, muscles, heart, brain, and kidneys [6, 7]. Dengue fever has been associated with various types of renal manifestations such as proteinuria, hematuria, glomerulonephritis, and acute kidney injury (AKI). The incidence of these renal manifestations varies between 17% and 62% in patients with dengue fever [8]. Such complications impose a heavy burden on the country not only in terms of morbidity and mortality but also impact the economic growth of the country. Currently, there is relatively sparse data from Bangladesh on the renal manifestations of dengue fever and their outcomes. Hence, this prospective cross sectional study was designed to analyze the frequency, characteristics, and clinical outcome of dengue fever in children with renal manifestations.

## 2. Methodology

This prospective cross sectional study was conducted in Dr. M R Khan Children Hospital and Institute of Child Health over a period of 1 year from January 2018 to December 2018. The study was approved by the ethical committee of the institute. All patients aged 6 months to 13 years who met the WHO criteria of dengue fever, i.e., clinical feature plus either NS1 positive or dengue IgM positive or both Dengue IgM and IgG positive patients were included in this study. Patients who did not give consent, who have preexisting renal disease were excluded from this study.

The disease severity was classified according to the World Health Organization criteria. The renal manifestations were divided into AKI and non-AKI groups using pRIFLE criteria which are described as follows. Proteinuria was defined as urinary protein >1+ (30 mg/dL) by dipstick test. Hematuria was defined as red blood cell (RBC) >5/*μ*L in a fresh uncentrifuged urine specimen. All patients were managed by hospital protocol and investigation was sent according to patient's condition and management protocol. So, we sent routine tests as well as electrolyte to see hematological and electrolyte changes among dengue patients for treatment purpose. These parameters also help for early identification of complications in study group.

For patients who had AKI with failure, peritoneal dialysis was initiated. Patients who need ICU support were shifted to ICU. Among the patient who had proteinuria and hematuria, none underwent renal biopsy at the time of diagnosis. They followed-up monthly for 3 months for any persisted significant proteinuria and hematuria for further biopsy plan. But no significant proteinuria or hematuria was found in any patient (Table 1).

## 3. Results

**Figure 1:**
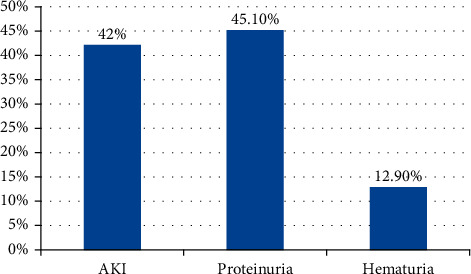
Types of renal involvement in children with dengue fever.

Among 316 dengue patients thirty-one patients (9.8%) had renal involvement. Among renal involvement, 14 patients (45.10%) had proteinuria, 13 (42%) had AKI, and 4 (12.90%) patients had hematuria (Figure 1). Among 4 patients of hematuria, 3 had only hematuria, and one had features of glomerulonephritis.

In dengue fever, only 3 (1.4%) patients were found to have renal involvement, whereas dengue shock syndrome and dengue hemorrhagic fever had highest renal involvement 35.8% and (17.9%), respectively (Table 2).

Table 3 showed mean serum creatinine (micromole/L) is significantly higher in patients with dengue fever (185.17 ± 121.35) with renal involvement vs. patient without renal involvement (85.50 ± 16.99) (*P* value <0.001) and mean hematocrit level is also significantly higher in the renal group (41.49 ± 4.47) than in patients without renal manifestation (39.98 ± 5.16) (*P* value <0.005). Serum Na^+^, serum K^+^, and platelet and leukocyte changes are not significant between two group.

Table 4 showed among 14 patients with proteinuria, 7 (50%) had mild proteinuria, 3 (21.4%) had moderate, 3(21.4%) patients had severe proteinuria, and only one patient had nephrotic-range proteinuria.

Among 13 patients with AKI in children with dengue fever, mostly 46.15% had risk, 30.76% had failure requiring peritoneal dialysis, and 23.07% had only injury managed conservatively (Table 5).

Table 6 showed in this study, among thirty-one patients with renal involvement, 19 had platelet count <50,000 with high mortality (2 patients died). Another patient died whose platelet count was 50,000–100,000. Mortality zero when platelet is more than 100,000.

The duration of hospital stay in patients with AKI was 6 ± 2 days in risk group, 7 ± 5 days in injury, and 11 ± 2 days in patients with failure group. Recovery of renal function was seen in all patients with pRIFLE risk and injury group and one patient with failure group (Table 7).

13 patients were found with AKI according to the pRIFLE criteria; out of 13 patients, 4 patients were in failure/had failure and required peritoneal dialysis. Three of them died. Among this 3 deaths, 2 had platelet count <50,000 and 1 had platelet count 50,000–100,000. The cause of death was multiorgan failure. Among these 3 deaths the duration of PD was 48–72 hours. One patient out of 4 patients with failure was recovered fully, and duration of dialysis was 72 hours.

Among thirty-one patients of renal involvement, 14 patients (45.10%) had proteinuria, 13 (42%) had AKI, and 4 (12.90%) patients had hematuria.

In dengue fever, only 3 (1.4%) patients were found to have renal involvement, whereas dengue shock syndrome and dengue hemorrhagic fever had highest renal involvement 35.8% and 17.9%, respectively.

Table 3 showed mean serum creatinine is significantly higher in patients with dengue fever with renal involvement (*P* value <0.001), and hematocrit level is significantly higher in the renal group than in patients without renal manifestation (*P* value <0.005). Serum Na^+^, serum K^+^, and platelet and leukocyte changes are not significant.

Among 14 patients with proteinuria 7 (50%) had mild proteinuria, 3 (21.4%) had moderate, 3 (21.4%) patients had severe proteinuria, and only one patient (7%) had nephrotic range of proteinuria.

Among thirty-one patients with renal involvement majority, 19 had platelet count<50,000 with high mortality (2 patients died). Another patient died whose platelet count was 50,000–100,000. Mortality was zero when platelet was more than 100,000.

The duration of hospital stay in patients with AKI was 6 ± 2 days in the risk group, 7 ± 5 days in injury, and 11 ± 2 days in patients with the failure group. Recovery of renal function was seen in all patients with pRIFLE risk and injury group and one patient with failure group.

## 4. Discussion

Dengue fever can be presented with variety of clinical presentation with unpredictable progression and outcomes. Its presentation ranges from asymptomatic forms to severe shock, eventually resulting in death [9].

Dengue infection has been associated with various renal disorders. Acute renal failure, proteinuria, hematuria, and glomerulonephritis have been reported during or shortly after acute dengue infection. In our study, among 316 dengue patients, thirty-one patients (9.8%) had renal involvement. Eswarappa et al.'s retrospective study showed prevalence of renal manifestation in dengue fever is 9.02% of the study population, which is similar to our study [10].

In this study, thirty-one patients of renal involvement majority (45.10%) had proteinuria. Among 14 patients with proteinuria mostly (50%) had mild proteinuria, 3 (21.4%) had moderate 3 (21.4%) patient had severe proteinuria, and only one pt had nephrotic range of proteinuria. Similar findings found in Horvath et al., where proteinuria in 74% of patient in whom urinalysis was performed during a dengue-3 epidemic in Queensland, Australia [11]. In this cohort, one patient had 10.8 g/day proteinuria and was diagnosed with the nephrotic syndrome. Vasanwala and colleagues reported two DHF patients with nephrotic-range proteinuria. Daily protein excretion was 8.1 g/day and 9.0 g/day based on a random urine protein-to-creatinine ratio. These patients did not have hematuria or elevated serum creatinine concentrations [12].

Whereas in our study, prevalence of proteinuria was 45.01%, and Garcia et al. retrospectively studied 74 patients with dengue fever or DHF who had a platelet count of less than 125,000/mm^3;^ the prevalence of proteinuria in this cohort was 30% [13]. This wide difference of prevalence 45.01% in our study to 74% and 30% may be due to regional epidemic occurrence, host response, and type of dengue virus strain.

In the present study, 13 patients (42%) who had AKI in children with dengue fever mostly (46.15%) had risk, 30.76% had failure requiring peritoneal dialysis, and 23.07% had only injury managed conservatively.

The reported frequency of AKI in dengue fever association exhibits wide variation in accordance with the particular population being assessed, severity of dengue, criteria used for the diagnosis of AKI, and time of evaluation. Laoprasopwattana et al. [14] reported an incidence of 0.9% among children in Thailand, and Lee et al. [15] reported an incidence of 3.3% among adults in Taiwan. In a Brazilian intensive care unit for infectious diseases, dengue was the cause of 4% of the cases of AKI diagnosed using the risk, injury, failure, loss of kidney function, and end-stage acute kidney disease (RIFLE) criteria [16]. In a more recent study that employed the Acute Kidney Injury Network (AKIN) criteria for diagnosis, the incidence of AKI was 10.8% [17]. Using the AKIN criteria in a retrospective analysis, Khalil et al. identified AKI in 13.3% of a series of patients with dengue confirmed by the presence of IgM antibodies, independent of the severity of disease; 64.8% of the patients were in stage 1, 18.3% stage 2, and 16.9% stage 3 of the disease [18] In another study, the RIFLE classification was used to investigate the occurrence of AKI in patients with tropical acute febrile disease. The results showed that the incidence of AKI among patients with dengue upon admission to the hospital was 35.7% [19]. Eswarappa et al. in another recent retrospective study found AKI in 82 patients (3.4%) using AKIN staging with AKIN stage I seen in 58 patients (70.73%) [10]. In the present study, we found 42% had AKI which is close to study of Basu et al where they found 35.7% [19]. This wide variation may be due to various risk factors for developing AKI with dengue infection. In our study, we found younger age, severe thrombocytopenia, shock, hypotension, fluid overload, pulmonary edema, and MODS are identifiable associated factors for developing AKI which is similar to a reviewed etiopathogenesis of AKI by Olivera and Burdmann where several mechanisms have been proposed to account for the etiopathogenesis of dengue fever-induced AKI, including direct action by the virus, hemodynamic instability, rhabdomyolysis, hemolysis, and acute glomerular injury [20].

In our study, 4 (12.90%) patients had hematuria which is similar to the study by Lizarraga et al, where both studies hematuria has been reported in up to 12.5% of patients with DHF [8, 21]

In dengue fever, only 3 (1.4%) patients were found to have renal involvement, whereas dengue shock syndrome and dengue hemorrhagic fever had highest renal involvement of 35.8% and 17.9%, respectively. These findings correlate with another study by Guzman et al. where they found dengue shock syndrome had the highest percentage (37.5%) of renal involvement [22].

Among thirty-one patients with renal involvement, 19 had platelet count<50,000 with high mortality (2 patients died). Another patient died whose platelet count was 50,000–100,000. Mortality was zero when platelet was more than 100,000.

In this study, mean serum creatinine (micromole/L) is significantly higher in patients with dengue fever (185.17 ± 121.35) with renal involvement vs. patients without renal involvement (85.50 ± 16.99) (*P* value <0.001), and hematocrit level is also significantly higher in the renal group (41.49 ± 4.47) than in patients without renal manifestation (39.98 ± 5.16) (*P* value <0.005). Serum Na^+^, serum K^+^, and platelet and leukocyte changes are not significant. Tauqeer et al. showed same findings in a retrospective study. They also found hematocrit rises significantly in AKI with dengue fever group than non-AKI dengue fever [23]. In another study, laboratory abnormalities found in patients with dengue include leukopenia (usually below 4000 cells/mm^3^) and relative lymphocytosis (60–80%) and in severe dengue, intense thrombocytopenia and increased hematocrit values. In addition, the patients might exhibit abnormal results on the coagulation tests, increased urea and creatinine levels, low complement component 3 (C3) levels, and alterations in urinalysis results (proteinuria, hematuria, and leukocyturia) [24].

The duration of hospital stay in patients with AKI was 6 ± 2 days in the risk group, 7 ± 5 days in injury, and 11 ± 2 days in patients with failure group. Recovery of renal function was seen in all patients with pRIFLE risk and injury group and one patient with failure group.

Retrospective studies of case series of dengue have shown that the development of AKI was associated with a longer hospital stay and higher mortality [10, 18, 22, 24–26] which is similar to our study.

Among 13 patients with AKI, 4 patients were in failure, 3 of them died, 2 had platelet count <50,000, and one had platelet count 50,000–10000. The cause of death was multiorgan failure. In a retrospective series, 60% of hospitalized DHF patients with ARF died. DHF patients with ARF were predominantly older men and had other comorbidities. Multivariate analysis showed that DSS was an independent risk factor for the development of ARF in patients with DHF [17].

## 5. Conclusion

Dengue fever is associated with a variety of renal disorders. Acute renal failure is a serious complication of dengue fever and carries a high mortality rate. Transient proteinuria and hematuria has been detected in most patients with dengue fever. These findings will draw an attention to the need for clinicians' alertness to this renal complication of dengue fever in children. Adequate knowledge of clinical profile and predictors of AKI development would help in early intervention to prevent complication of renal involvement in children with dengue fever.

## Figures and Tables

**Table 1:** The pediatric RIFLE (pRIFLE) criteria adapted for defining the stage of AKI in children with dengue fever.

pRIFLE	Creatinine	Urine output criteria (ml/kg/hr)
Risk	Increased creatinine 1.5 times from baseline or eGFR decreased by 25%	<0.5 for 8 hours

Injury	Increased creatinine 2 times from baseline or eGFR decreased b >50%	<0.5 for 16 hours

Failure	Increased creatinine 3 times from baseline or eGFR decreased b >75%	<0.3 for 24 hours or anuria for 12 hours

Loss	Persistent failure >4 weeks	

End stage	Persistent failure >3 months	

^*∗*^Acute Dialysis Quality Initiative group created the RIFLE criteria in 2004, and the pediatric RIFLE (pRIFLE) criteria adapted RIFLE criteria for use in children.

**Table 2:** Renal involvement according to severity of dengue.

Variable	Total no. of patients (*n* = 316)	Patients with renal involvement (*n* = 31)
Dengue fever	210	3 (1.4%)
Dengue haemorrhagic fever	67	12 (17.9%)
Dengue shock syndrome	39	14 (35.8%)

**Table 3:** Comparison of laboratory characteristics among dengue patients with and without renal involvement.

Parameters	Patients with renal involvement (*n* = 31)	Patients without renal involvement (*n* = 285)	*P* value
Serum creatinine (*μ*mol/L, mean ± SD)	185.17 ± 121.35	85.50 ± 16.99	<0.001
Serum Na (mmol/L, mean ± SD)	135.95 ± 7.18	135.28 ± 7.27	0.679 NS
Serum K (mmol/L, mean ± SD)	3.93 ± 0.59	3.73 ± 0.55	0.112 NS
Leucocytes (×10^9^/L, mean ± SD)	5.10 ± 4.25	5.13 ± 13.83	0.918 NS
Platelet (×10^9^/L, mean ± SD	85.51 ± 48.29	93.89 ± 65.15	0.196 NS
Hematocrit (%/L, mean ± SD)	41.49 ± 4.47	39.98 ± 5.16	0.005^*∗*^

^*∗*^Calculated by student-*t* test or Mann–Whitney *U* test, where appropriate.

**Table 4:** Types of proteinuria in patients with dengue fever.

Protienuria	Number of patients (%)
+	7 (50%)
++	3 (21.4%)
+++	3 (21.4%)
Nephrotic range	1 (7%)
Total	14 (100%)

**Table 5:** AKI in children with dengue fever. Among 13 patients with AKI in children with dengue fever, mostly 46.15% had risk, and 23.07% had only injury managed conservatively. 30.76% had failure requiring peritoneal dialysis.

pRIFLE criteria	Number of patients (%)
Risk	6 (46.15%)
Injury	3 (23.07%)
Failure	4 (30.76%)
Total	13

**Table 6:** Outcome of renal involvement associated with platelet count.

Platelets (per *μ*L)	Total patients with renal involvement (*n* = 31)	No mortality (*n* = 28)	Mortality (*n* = 3)
<50,000	19	17	2
50,000–100,000	8	7	1
100,000–150,000	3	3	0
>150,000	1	1	0

**Table 7:** Duration of hospital stay in children with AKI.

pRIFLE criteria of AKI	Hospital stay in days (mean ± SD)
Risk	6 ± 2
Injury	7 ± 5
Failure	11 ± 2

## Data Availability

The data used to support the findings of this study are available from the corresponding author upon request.

## Additional Points

(i) This is a single center study and (ii) the sample size is small.
